# Management of orbital invasion in esthesioneuroblastoma: 14 years’ experience

**DOI:** 10.1186/s13014-019-1313-1

**Published:** 2019-06-13

**Authors:** Ruichen Li, Shu Tian, Yi Zhu, Li Yan, Wenjia Zhu, Huatao Quan, Shengzi Wang

**Affiliations:** 1 Department of Radiation Oncology, Eye & ENT Hospital, Fudan University, 83 Fenyang Road, Xuhui, Shanghai, 200031 People’s Republic of China; 2 Department of E.N.T, Eye & ENT Hospital, Fudan University, Shanghai, 200031 China

**Keywords:** Esthesioneuroblastoma, Orbital invasion, Orbital preservation, Radiotherapy, Prognostic factors

## Abstract

**Background:**

There is a scarcity of data about the prognostic value of orbital invasion in esthesioneuroblastoma (ENB), as well as about its management strategies. Indications for the preservation of orbital contents remain controversial, and the evaluation of orbital invasion has been ill defined.

**Methods:**

This retrospective analysis contained 60 ENB patients with orbital invasion who underwent radiotherapy with or without surgery over the past 14 years. Orbital invasion was classified into three grades.

**Results:**

There were 52 patients at stage C and 8 at stage D, according to Foote classifications. Grade I, grade II and grade III orbital invasion was detected in 12, 23, and 25 patients, respectively. The median follow-up was 57 months (IQR 32–95 months). Fourteen patients received radical radiotherapy, with a 5-year overall survival (OS) of 63.5%; 46 received surgery plus radiation, with a 5-year OS of 70.7%; and the difference was not statistically significant (*p* = 0.847). Orbital preservation was feasible in 100% of cases, including 18 cases that extended to extraocular muscles or the eye globe. Five-year locoregional relapse-free survival was 100% in patients with prophylactic elective neck irradiation (PENI) and 58.1% in patients without PENI (*p* = 0.004). Univariate analysis showed that grade II/III orbital invasion was associated with poorer OS and progression-free survival. Neck metastasis (with a Foote stage of D) was independently associated with shorter OS and distant metastasis–free survival in multivariate analysis.

**Conclusions:**

Our data suggested that primary radiotherapy achieved comparable survival to surgery plus radiotherapy in advanced ENB. Invasion of either the extraocular muscles or the eye globe is not a contraindication for eye-sparing surgery. Orbital invasion in grade II/III was significantly associated with adverse survival outcomes. Prophylactic radiotherapy to the neck with N0 significantly reduces the risk of regional recurrence.

## Introduction

Esthesioneuroblastoma (ENB) is a rare malignant tumor of the nasal vault that is usually diagnosed at a locally advanced stage [1]. Orbital invasion is frequently observed in ENB involving the ethmoid sinus. It has been proven that orbital invasion is associated with a poor prognosis and is commonly evaluated as stage C disease, according to the Kadish/Foote staging system [2]. The prognostic value of the distinction between invasion of the bony orbit and of the deeper soft tissue has been reflected in the American Joint Committee on Cancer (AJCC) staging categories, which divide ethmoid and maxillary tumors and differentiate the role of bone orbital wall invasion (T3) from invasion of the anterior two-thirds (T4a) and orbit apex (T4b), at least for squamous cell carcinoma and adenocarcinoma [3, 4]. However, whether the prognostic value of the degree of orbital invasion is applicable to ENB remains an open question due to the rarity of ENB.

The periorbit plays an important role against invasion and is recognized as a “landmark” of orbital exenteration over time in sinonasal tumors [5–7]. With the introduction of endoscopic surgery and advanced radiotherapy techniques [8], the indications for orbital exenteration are continuously changing. Agreement has not been reached on the degree of orbital invasion that is oncologically safe when sparing orbital contents. A recent study had showed that orbital preservation could be performed in cases of tumors not adherent to extraocular muscles [9]. However, the histology of sinonasal malignancies was extremely heterogeneous in previous studies [9–11], and the evaluation of orbital invasion was unclear [11], thus raising the risk of statistical bias. Furthermore, the indication for orbital exenteration in squamous cell carcinoma or adenocarcinoma may not be generalizable to ENB due to their differences in biological characteristic and prognosis.

We believe that orbital preservation can be achieved with the assistance of radiotherapy, even if the extraocular muscles and eye globe are involved in ENB. In the current study, we retrospectively reviewed and summarized our therapeutic strategy over the past 14 years for 60 ENB patients with orbital invasion to clarify the specific degree of orbital invasion and to analyze its influence on survival and orbital preservation. To the best of our knowledge, this is the largest study in which treatment and prognostic factors have been assessed in patients with ENB invading the orbit.

## Material and methods

### Patient characteristics

Between April 2004 and August 2017, 60 ENB patients with orbital invasion received treatment at the Eye & ENT Hospital, Fudan University, China, all of whom underwent radiotherapy. The exclusion criteria were as follows: 1. incomplete follow-up information or lost to follow-up; 2. insufficient medical history; 3. evidence of distant metastasis; 4. without orbital invasion; 5. history of other cancers; 6. receipt of palliative treatment. All of the patients underwent a radiological examination (MRI or high-resolution and contrast-enhanced CT scan or both) to evaluate the location and extent of the tumor. We classified orbital involvement into the following three grades [12]: grade I, bone wall invasion; grade II, invasion of extraconal fat; and grade III, involvement of extraocular muscles, eye globe, orbital apex, or optic nerve. All the cases were staged according to the Foote classifications (modified version of Kadish staging system). The clinical information of the study population is shown in Table 1.

**Table 1:** Clinical characteristics and treatment methods of evaluable patients

Variable	No. (%)
Median age, yr. (range)	54 (22–77)
Sex	
Male	44 (73.3%)
Female	16 (26.7%)
Degree of orbital invasion^a^	
Grade I	12 (20%)
Grade II	23 (38.3%)
Grade III	25 (41.7%)
Foote stage	
C	52 (86.7%)
D	8 (13.3%)
Lymph node classification	
N0	52 (86.7%)
N+	8 (13.3%)
Orbital symptoms	
Yes	41 (68.3%)
No	19 (31.7%)
Treatment methods	
Radical radiation	14 (23.3%)
Preoperative radiation + surgery	21 (35%)
Postoperative radiation +surgery	25 (41.7%)
Surgical methods	
Endoscopic group	33 (71.7%)
Open group^b^	13 (28.3%)
Management of neck	
PENI	25 (41.6%)
TENI	7 (11.7%)
END	1 (1.7%)
No management	27 (45%)
Type of radiotherapy	
3D-CRT	34 (56.7%)
IMRT	26 (43.3%)
Chemotherapy	
Yes	47 (78.3%)
No	13 (21.7%)

*Abbreviation*: *3D-CRT* three-dimensional conformal radiotherapy, *IMRT* intensity-modulated radiotherapy, *PENI* prophylactic elective neck irradiation, *TENI* therapeutic elective neck irradiation, *END* elective neck dissection

^a^Grade I, bone wall erosion; grade II, invasion of extraconal fat; grade III, involvement of extraocular muscles, eye globe, orbital apex, or optic nerve

^b^Open group included lateral rhinotomy and craniofacial resection

### Treatment data

A strategy of preoperative radiation was applied to cases of advanced tumors, for which surgical treatment is challenging in terms of performing radical surgery while preserving organs. For those cases at high risk of recurrence after surgery, including those with gross residual disease, positive surgical margins, and a Foote stage of C/D, postoperative radiation was performed. Patients with unresectable disease, a poor condition that precluded major surgery or a refusal to undergo surgery were treated with radical radiation. Forty-seven patients received platinum-based chemotherapy.

All patients had a strong desire to preserve their eyes. The surgical methods were classified as either endoscopic or open (i.e., lateral rhinotomy or craniofacial resection). Elective neck dissection was performed on one N+ patient. All patients were irradiated using three-dimensional conformal radiotherapy (3D-CRT) or intensity-modulated radiotherapy (IMRT). The patients were immobilized with a thermoplastic mask during the treatment. A set of CT images from the head to the clavicle (slice thickness of 2.5 mm for the primary lesion and 5 mm for the neck) was obtained for treatment planning. MRI was performed to identify intracranial extension if skull base bone invasion was revealed by CT. All plans were generated by a Treatment Planning System (ADAC Pinnacle 3, version 7.0, Varian Medical Systems, Palo Alto, CA). Determination of the gross tumor volume (GTV) was based on the results of MRI or a CT scan. The clinical target volume (CTV) was defined as the GTV plus a margin of 5–10 mm, which included high-risk CTV and low-risk CTV. The planning target volume (PTV) had a margin of 3 mm (IMRT) or 5 mm (3D-CRT) added around the CTV, and the margin was reduced in areas where the volume was adjacent to critical normal structures. The average irradiation dose for the patients with definitive radiotherapy, preoperative radiation, and postoperative radiation was 67.66 Gy (63.6–78 Gy), 63.53 Gy (50–71.9 Gy) and 62.79 Gy (50–68.9 Gy), respectively. Seven patients with N+ received ipsilateral therapeutic elective neck irradiation (TENI), with an average irradiation dose of 64.5 Gy (60–68.2 Gy); of 52 N0 patients, 25 patients were treated with prophylactic elective neck irradiation (PENI), with an average irradiation dose of 53.6 Gy (40–57 Gy), while 27 were not. The dose constraints for organs at risk were as follows: spinal cord (≤ 45 Gy), optic nerve (≤ 54 Gy), optic chiasm (≤ 54 Gy), and brainstem (≤ 54 Gy). Dose-volume histograms were retrieved from the planning system and used for planning evaluation. The treatment plans were normalized to cover 95% of the PTVs with the prescribed dose.

### Follow-up

The treatment efficacy was evaluated based on the results of a radiological examination and Response Evaluation Criteria in Solid Tumors (version 1.1). Adverse reactions were graded according to the scoring system of the Radiation Therapy and Oncology Group. Follow-up information was obtained from outpatient reviews and telephone interviews. The last investigation was held in August 2018, and the median follow–up duration was 57 months (IQR 32–95 months).

### Statistical analysis

The Kaplan-Meier method was used to estimate the probability of locoregional relapse-free survival (LRFS), distant metastasis–free survival (DMFS), progression-free survival (PFS), and overall survival (OS). Locoregional relapse is defined as follows: recurrence of primary site or cervical lymph node metastasis. Distant metastasis is defined as follows: metastasis outside primary site and cervical lymph node, such as lung, bone or liver. Overall survival is defined as follows: death by any cause. Progression is defined as follows: progression, recurrence, distant metastasis or death by any cause. The survival was calculated from the date of the first administration of therapy to the date of the event or last follow-up. The log-rank test and Cox hazard model (forward approach) were used in univariate and multivariate analyses, respectively. Variables with *p* < 0.05 in the univariate analyses were included in the multivariate analyses. Statistical analyses were performed using SPSS, version 22 (SPSS, Chicago, IL). For all analyses, the *p* values were two-sided, and *p* < 0.05 was considered statistically significant.

## Results

### Clinical features

A total of 60 patients were included in this study, which consisted of 44 males (73.3%) and 16 females (26.7%), with a median age of 54 years (range = 22–77 years). The basic clinical information is presented in Table 1. Twelve patients (20%) had grade I orbital invasion, 23 (38.3%) had grade II orbital invasion, and 25 (41.7%) had grade III orbital invasion. All primary tumors were located in the nasal cavity with (*n* = 8) or without lymph node metastasis (*n* = 52). According to the Foote staging system (modified version of Kadish staging system), the distribution of patients with stage C and stage D ENB was 52 (86.7%) and 8 (13.3%), respectively. Most patients had orbital symptoms (68.3%). We performed surgical resection with orbital preservation in 46 patients (100%), and no patient underwent orbital exenteration.

### Survival outcomes in different treatment modalities

**Fig. 1:**
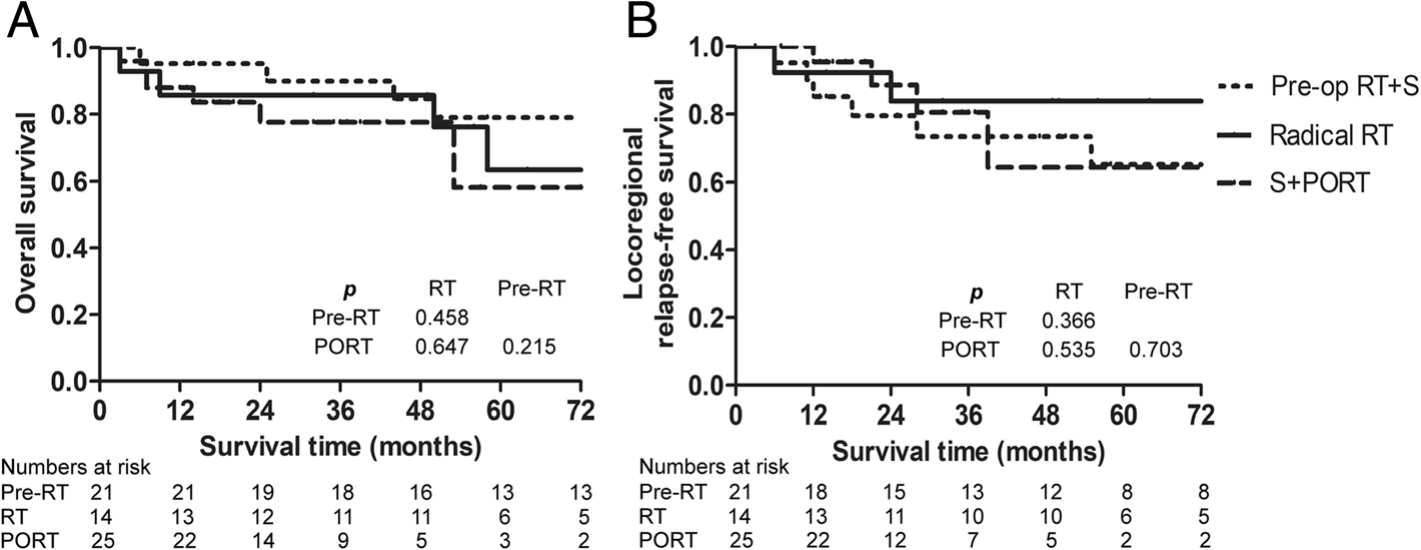
Survival curves among various treatment modalities. **a** Overall survival; **b** Locoregional relapse free survival

Forty-six patients received surgery plus radiation with or without chemotherapy, whereas 14 patients received definitive radiotherapy with or without chemotherapy. As depicted in Fig. 1, there was no significant difference in the survival curves treated with or without surgery (*p* = 0.847). Patients treated with surgery had a 5-year OS of 70.7%, compared to 63.5% in the nonsurgically treated patients. There was also no significant difference in 5-year LRFS (66.3% vs. 83.9%, *p* = 0.407).

**Fig. 2:**
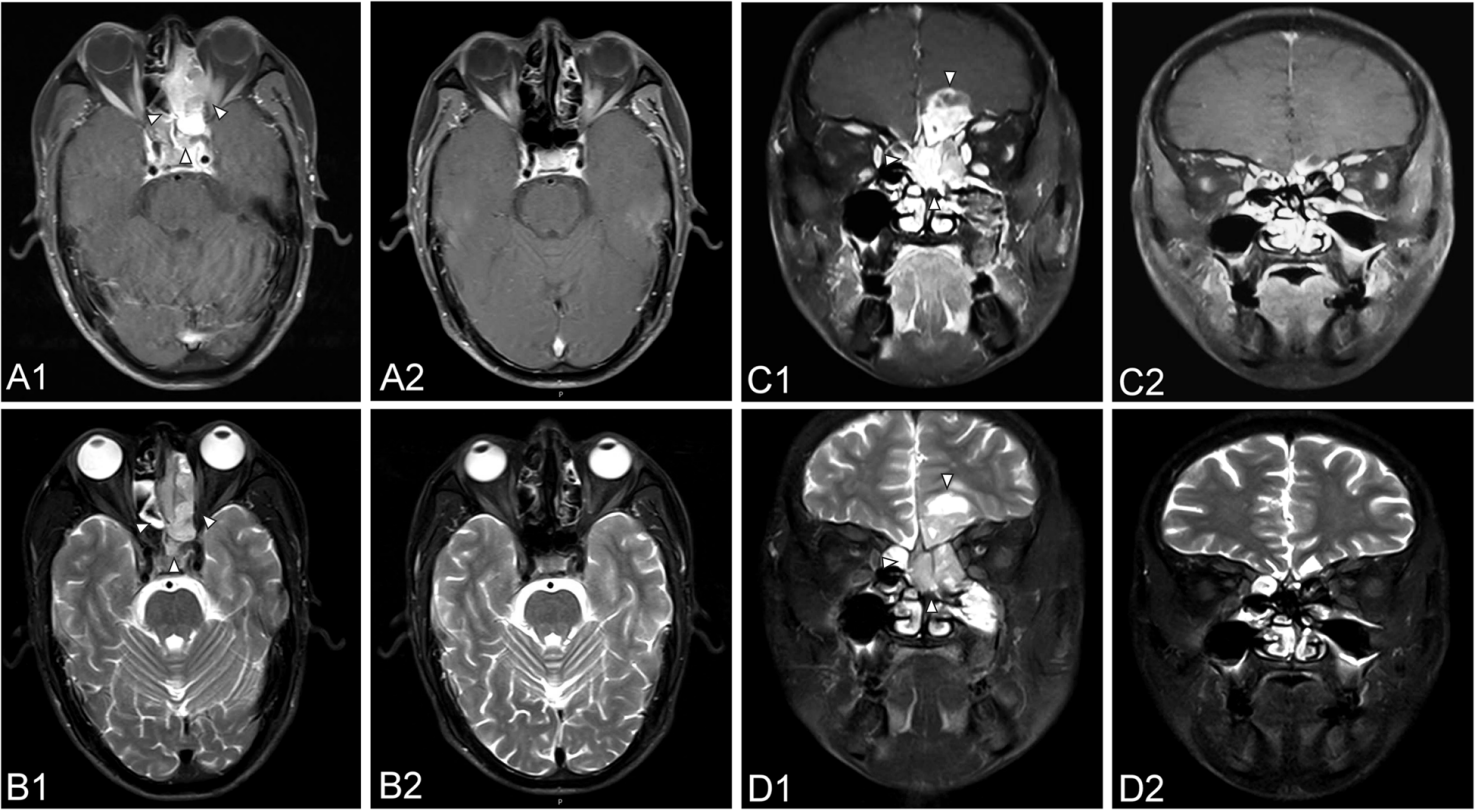
Comparison of MRI before and after treatment for a 50-year-old patient with ENB (stage of C) who received definitive radiotherapy without surgery. (**A**) and (**B**) refer to axial MRI, (**C**) and (**D**) refer to coronal MRI. 1, 2 refer to MRI before treatment, after radiotherapy, respectively. MRI before treatment revealed a large mass on the left nasal cavity and ethmoid sinus, which invaded the extraocular muscle of the left orbit (**A1** arrowhead, **B1** arrowhead). The tumor extended to beyond the midline, left frontal sinus and sphenoid sinus, and frontal lobe (**C1** arrowhead, **D1** arrowhead). After radiotherapy, the patient had a complete response

The average irradiation dose among the patients with definitive radiotherapy was 67.66 Gy (63.6–78 Gy). After treatment, 7 (50%) patients had a complete response (CR), 6 (42.9%) had a partial response (PR), and 1 (7.1%) had progression of the disease (PD). Among the 7 patients with CR, one patient died of cardiovascular disease, with a survival time of 58 months, after experiencing cervical recurrence three times; another patient died of distant metastasis, with a survival time of 50 months. The remaining 5 patients were disease-free and still alive at the end of follow-up. Figure 2 shows a comparison of the MRI before and after treatment in one typical case, which had invaded to extraocular muscle. To date, this patient had been followed up for 50 months with no recurrence. Among the 6 patients with PR, 3 patients showed abnormal signals of residual tumors, which reduced gradually and disappeared spontaneously during follow-up. Another 2 patients showed no progression of the disease. The remaining patient underwent additional surgery but died of brain metastasis, with a survival time of 9 months. The patient with PD died after a survival time of 3 months.

**Fig. 3:**
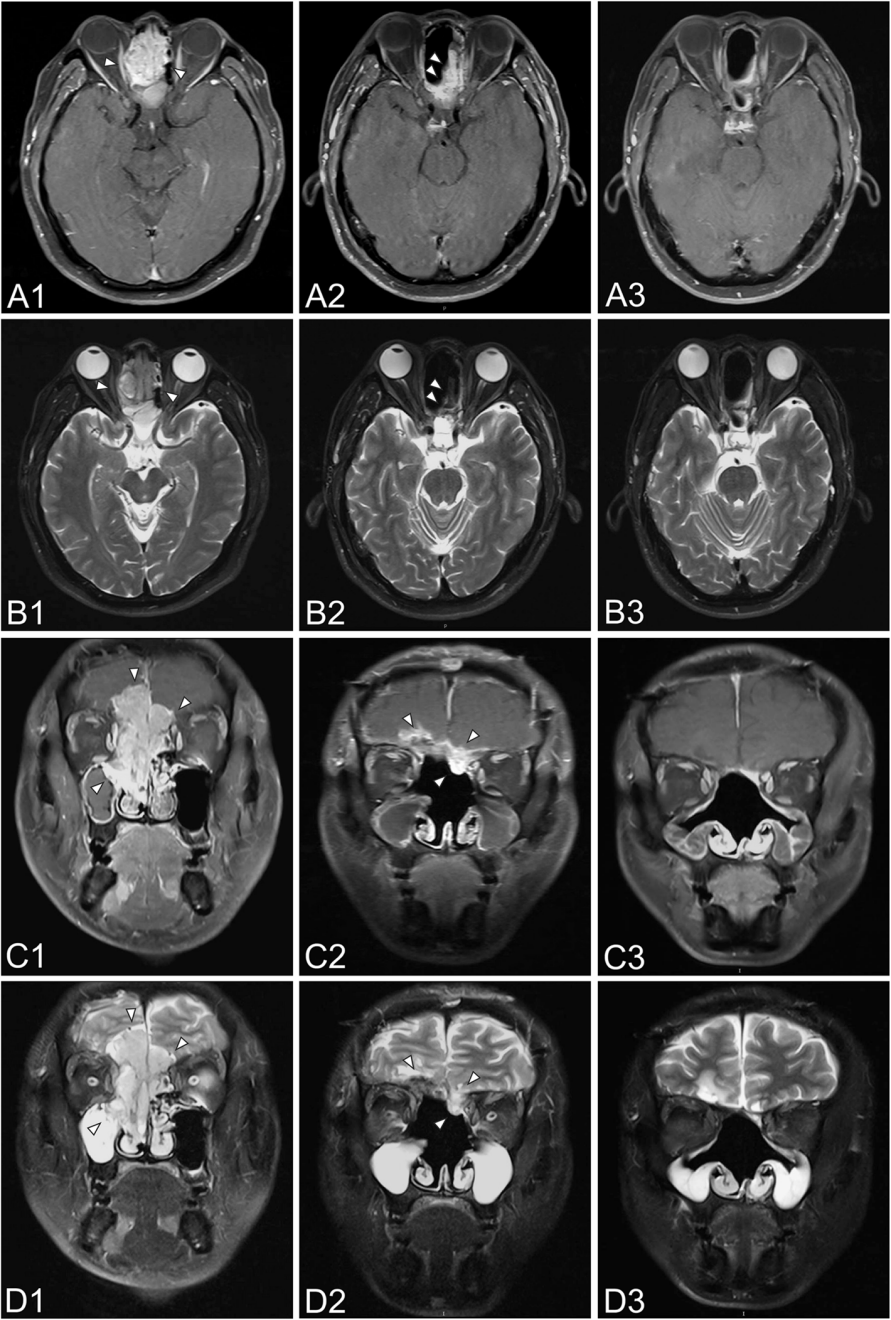
Comparison of MRI before and after treatment for a 38-year-old patient with ENB (stage of C) who received surgery plus postoperative radiation. (**A**) and (**B**) refer to axial MRI, and (**C**) and (**D**) refer to coronal MRI. 1, 2, and 3 refer to MRI before treatment, after surgery, and after radiotherapy, respectively. MRI before treatment revealed a large mass on bilateral nasal cavity and ethmoid sinus, which invaded the extraconal fat of the right orbit (**A1** arrowhead, **B1** arrowhead). The tumor extended to right maxillary sinus and sphenoid sinus, bilateral anterior skull base and frontal lobe (**C1** arrowhead, **D1** arrowhead). After surgery, residual tumor was observed at the site of ethmoid sinus (**A2** arrowhead, **B2** arrowhead), bilateral anterior skull base and frontal lobe (**C2** arrowhead, **D2** arrowhead). After postoperative radiation, the residual tumor disappeared

Of the 46 patients who underwent surgery, 21 cases received preoperative radiation and 25 cases received postoperative radiation. The median times between surgery and radiation in pre- and postoperative radiation subgroups were 35 days (12–78 days) and 27 days (13–43 days), respectively. The average dose was 63.53 Gy (50–71.9 Gy) and 62.79 Gy (50–68.9 Gy), respectively. The 5-year OS was 79% for preoperative radiation and 58.2% for postoperative radiation (Fig. 1), although the difference was not statistically significant (*p* = 0.215). Among the 25 patients with postoperative radiation, residual tumors were found in 19 cases (76%) after surgery. The patients without residual tumors had a 5-year OS of 100%, which fell to 53.1% in patients with residual tumors (*p* = 0.187), and a similar relationship was shown in the 5-year PFS for the two groups (100% vs. 42.8%, respectively, *p* = 0.088). As depicted in Fig. 3, after primary surgery, a residual tumor was observed in MRI. Postoperative radiation was delivered later, and the residual tumor disappeared. To date, this patient has been followed for 37 months with no recurrence. Treatment efficacy among the preoperative radiation group was as follows: 6 cases (28.6%) had CRs, 11 cases (52.4%) had PRs, and 4 cases (19%) had stable disease (SD). There was no difference in 5-year OS between CRs and non-CRs (80% vs. 78.3%, *p* = 0.941), nor the 5-year LRFS (66.7% vs. 66.3%, *p* = 0.521). In terms of radiotherapy and different surgical methods, the average irradiation dose in endoscopic group and open group was 63.62 Gy (56–68.8 Gy) and 61.87 Gy (50–71.9 Gy) (*p* = 0.607), respectively. Of the 46 patients, 5 patients (10.9%) experienced a local recurrence, and 8 patients (17.4%) experienced a regional recurrence.

**Fig. 4:**
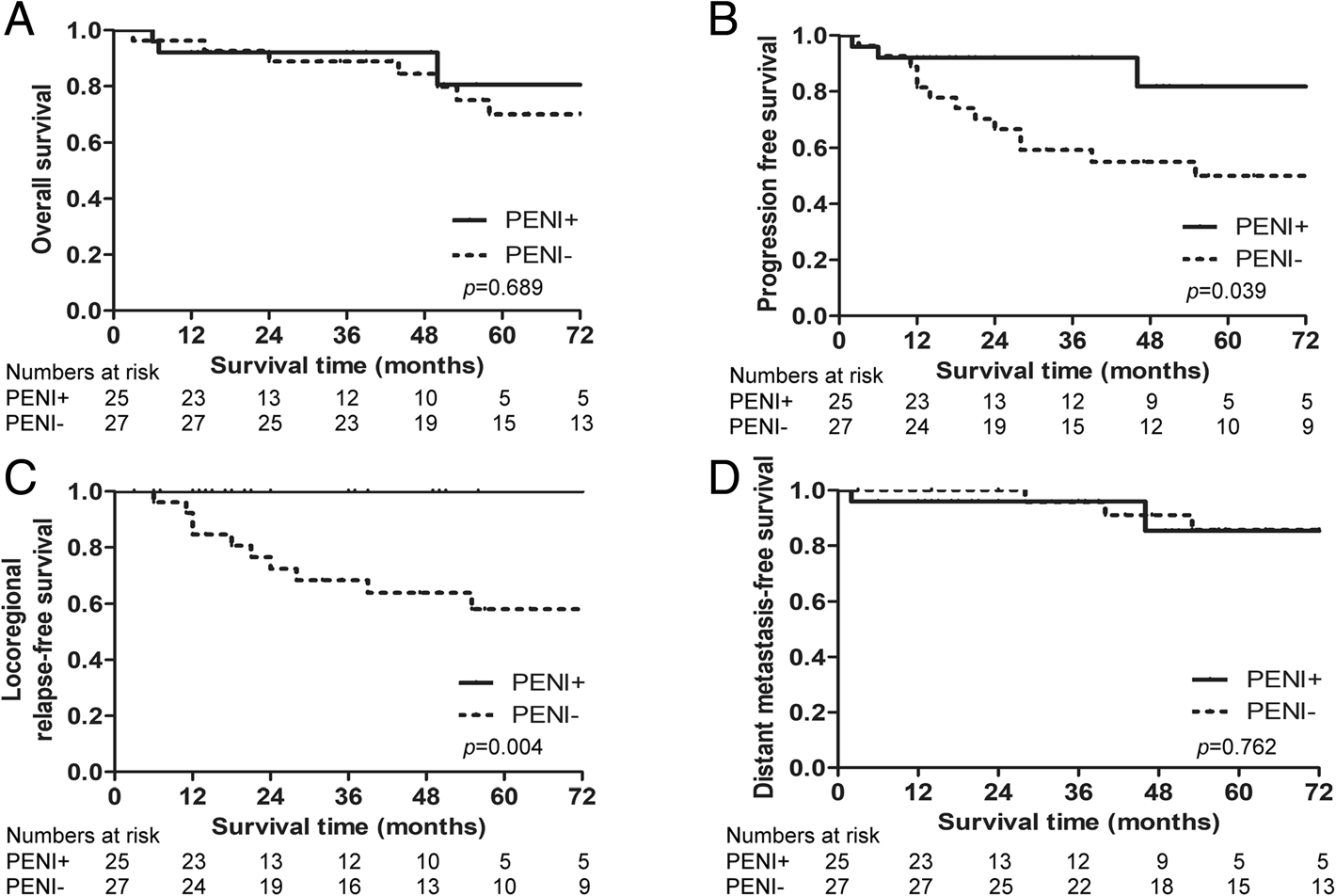
Survival curves for 52 patients treated with prophylactic elective neck irradiation (PENI)/non-PENI. **a** Overall survival; **b** Progression free survival; **c** Locoregional relapse free survival; **d** Distant metastasis free survival

Of the 8 patients who had clinical/radiographic evidence of neck metastasis (N+), 7 patients had undergone definitive radiotherapy of the neck (TENI) with no regional recurrence during the follow-up period. The remaining one, who was treated with elective neck dissection, had developed neck node recurrence 28 months after treatment and underwent neck dissection again. Of the 52 patients who were N0 at the time of initial treatment, 27 patients were treated without any form of PENI, while 25 patients were treated with PENI with an average irradiation dose of 53.6 Gy (40–57 Gy). Among the 27 cases who did not receive PENI, 8 (29.6%) developed node failures, compared with 0 of 25 patients (0%) who did receive PENI. The 5-year LRFS was 100% in patients with PENI and 58.1% in patients without PENI (*p* = 0.004), the 5-year DMFS was 85.3% in patients with PENI and 85.7% in patients without PENI (*p* = 0.762), the 5-year PFS was 81.8% in patients with PENI and 50% in patients without PENI (*p* = 0.039), and the 5-year OS was 80.5% in patients with PENI and 70.1% in patients without PENI (*p* = 0.689) (Fig. 4).

### Organ preservation and adverse reactions of normal tissues

**Fig. 5:**
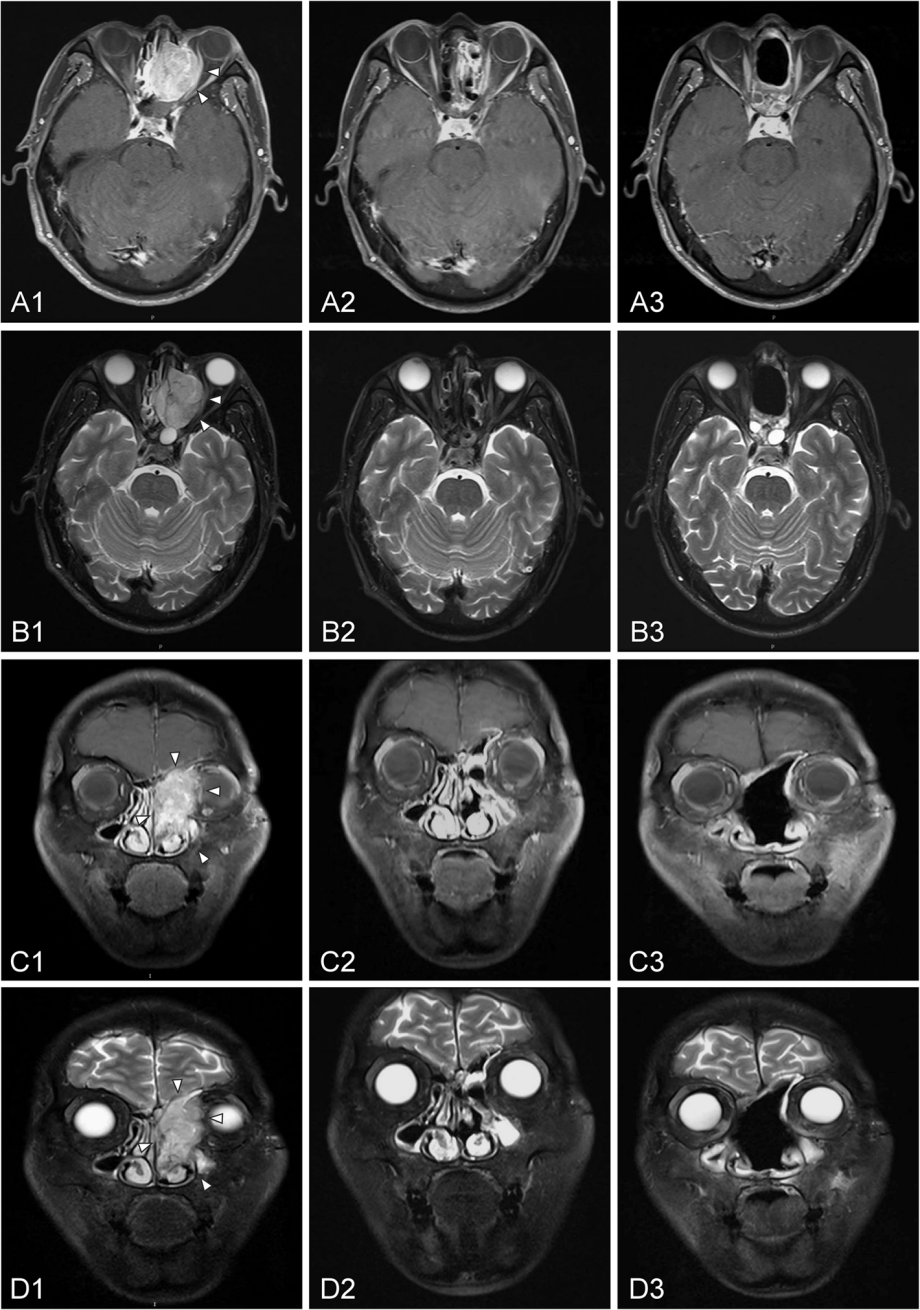
Comparison of MRI before and after treatment for a 43-year-old patient with ENB (stage of C) who received preoperative radiation plus surgery. (**A**) and (**B**) refer to axial MRI, and (**C**) and (**D**) refer to coronal MRI. 1, 2, and 3 refer to MRI before treatment, after preoperative radiation, and after surgery, respectively. MRI before treatment revealed a large mass on left nasal cavity and ethmoid sinus, which invaded the extraocular muscle of the left orbit (**A1** arrowhead, **B1** arrowhead). The tumor extended to left anterior skull base (**C1** arrowhead, **D1** arrowhead)

We performed surgical resection with orbital preservation in 46 patients (100%), including 18 cases that extended to extraocular muscles or the eye globe, and no patient underwent orbital exenteration. Figure 5 shows a comparison before and after preoperative radiation in one typical case that extended to extraocular muscle. After radiation, the tumor size shrank obviously and achieved nearly a complete response, thus demonstrating the feasibility of orbital preservation. To date, this patient has been followed for 51 months with no recurrence.

Regarding acute skin and eye reactions, most patients presented with grade 1 reactions, although grade 2 skin, mucosal, and eye reactions were observed in 3, 18, and 8 patients, respectively. No grade 3 or grade 4 reactions were observed. Late toxicity was assessed in 46 surviving patients. The most common vision-related side effect was dry eye syndrome, with an incidence of 39.1% (18 in 46 cases). With regard to visual impairment, three patients developed lateral visual loss (grade 4), and 2 patients developed grade 3 visual impairment. The causes of blindness were optic neuropathy in two patients and retinopathy in one patient. Cataract formation was successfully treated with surgery in 5 patients.

### Prognostic analysis

The median time to recurrence was 19.5 months (range = 6–55 months). During the follow-up period, 6 patients experienced local recurrences, and 9 patients experienced neck node recurrences. The 5-year LRFS was 72.5%. Distant metastases were recorded in 8 patients, and the 5-year DMFS was 80.9%. The univariate analysis of factors predictive of locoregional control showed that treatment without chemotherapy and non-PENI were significantly associated with reduced LRFS (Table 2). The univariate and multivariate analyses of factors predictive of DMFS showed that neck metastasis (with a Foote stage of D) were significantly correlated with adverse outcomes (hazard ratio [HR] = 4.713, *p* = 0.042). (Table 3).

**Table 2:** Univariate analysis predicting OS, PFS, LRFS, DMFS

Variable	5-year OS		5-year PFS		5-year LRFS		5-year DMFS	
	%	*p*	%	*p*	%	*p*	%	*p*
Age								
> 55	76.1	0.201	65	0.596	74.8	0.877	86.8	0.371
≤ 55	61.9		48.8		68		72.7	
Sex								
Male	70.4	0.461	56.4	0.739	65.9	0.168	84	0.101
Female	64		58.3		93.8		70	
Degree of orbital invasion^a^								
Grade I	100	0.043^*^	91.7	0.041^*^	91.7	0.223	100	0.121
Grade II/III	61.4		49.2		66.8		75.7	
Foote stage								
C	72.3	0.026^*^	61.7	0.046^*^	73.9	0.449	84.6	0.009^*^
D	50		37.5		64.3		57.1	
Lymph node classification								
N0	72.3	0.026^*^	61.7	0.046^*^	73.9	0.449	84.6	0.009^*^
N+	50		37.5		64.3		57.1	
Invasion of orbital apex or optic nerve								
Yes	62.3	0.173	53	0.154	80.8	0.849	80.8	0.387
No	70.2		59.4		71.4		81.5	
Chemotherapy								
Yes	65.5	0.335	63.9	0.216	80.3	0.016^*^	78.4	0.372
No	80.2		37.9		50.5		87.5	
Surgical methods								
Endoscopic group	73.8	0.796	54.5	0.358	68.1	0.268	84.6	0.8
Open group^b^	67.7		46.2		59.2		80	
Treatment methods								
Radical radiation	63.5	0.847	69.3	0.394	83.9	0.407	83.1	0.885
Radiation + surgery	70.7		53.8		66.3		80.2	
Type of radiotherapy								
3D-CRT	60.2	0.077	46.9	0.032^*^	67.7	0.277	71.6	0.028^*^
IMRT	89.7		79.6		82.8		100	
PENI								
Yes	80.5	0.689	81.8	0.039^*^	100	0.004^*^	85.3	0.762
No	70.1		50		58.1		85.7	

*Abbreviation*: *OS* overall survival, *PFS* progression free survival, *LRFS* locoregional relapse free survival, *DMFS* distant metastasis free survival, *3D-CRT* three-dimensional conformal radiotherapy, *IMRT* intensity-modulated radiotherapy, *PENI* prophylactic elective neck irradiation

^*^Statistically significant, *p* < 0.05

^a^Grade I, bone wall erosion; grade II, invasion of extraconal fat; grade III, involvement of extraocular muscles, eye globe, orbital apex, or optic nerve

^b^Open group included lateral rhinotomy and craniofacial resection

**Table 3:** Multivariate Cox regression analysis of OS, PFS, LRFS, DMFS

Variable	OS		PFS		LRFS		DMFS	
	HR (95% CI)	*p*	HR (95% CI)	*p*	HR (95% CI)	*p*	HR (95% CI)	*p*
Degree of orbital invasion^a^					Not included		Not included	
Grade II/III vs. Grade I	–	0.971	–	0.076				
Foote stage					Not included			
D vs. C	4.419 (1.362–14.345)	0.013^*^	–	0.07			4.713 (1.059–20.98)	0.042^*^
Chemotherapy	Not included		Not included				Not included	
No vs. Yes					3.519 (1.133–10.926)	0.03^*^		
Type of radiotherapy	Not included				Not included			
IMRT vs. 3D-CRT			0.323 (0.108–0.963)	0.043^*^			–	0.945

*Abbreviation*: *OS* overall survival, *PFS* progression free survival, *LRFS* locoregional relapse free survival, *DMFS* distant metastasis free survival, *HR* hazard ratio, *CI* confidence interval, *3D-CRT* three-dimensional conformal radiotherapy, *IMRT* intensity-modulated radiotherapy

^*^Statistically significant, *p* < 0.05

^a^Grade I, bone wall erosion; grade II, invasion of extraconal fat; grade III, involvement of extraocular muscles, eye globe, orbital apex, or optic nerve

**Fig. 6:**
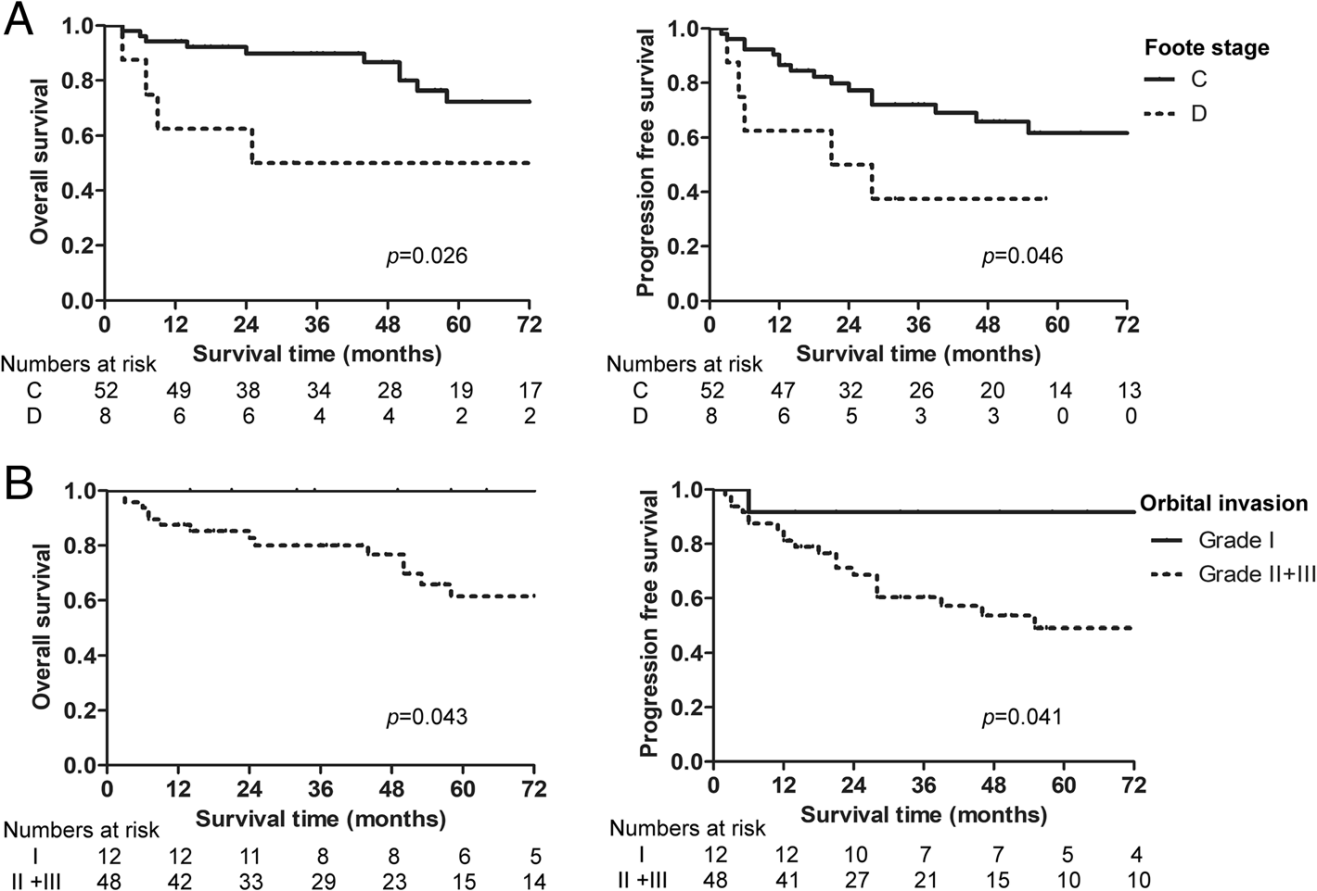
Kaplan-Meier OS (left) and PFS (right) curves for patients with ENB stratified by various clinical factors. **a** Survival curves for patients stratified by Foote stage; **b** for patients stratified by orbital invasion

By the end of the follow-up, 14 patients had died, with a 5-year OS of 69% and a 5-year PFS of 57.7%. The results of the univariate analysis showed that grade II/III orbital invasion and a Foote stage of D (neck metastasis) had significant effects on OS (Table 2). Univariate analysis showed that grade II/III orbital invasion, a Foote stage of D (neck metastasis), radiotherapy of 3D-CRT, and treatment without PENI were associated with reduced PFS. As depicted in Fig. 6, grade I orbital invasion was associated with better OS and PFS than was grade II/III. The 5-year OS was 100% in patients with grade I orbital invasion and 61.4% in patients with grade II/III orbital invasion (*p* = 0.043). The 5-year OS was 72.3% for a Foote stage of C, compared to 50% for a Foote stage of D (*p* = 0.026). In the multivariate analysis, a Foote stage of D (HR = 4.419, *p* = 0.013) was significantly predictive of shorter OS (Table 3). Radiotherapy of IMRT (HR = 0.323, *p* = 0.043) was an independently significant good prognostic factor for PFS.

## Discussion

Due to the rarity of ENB, there is limited data on this cancer in the literature. As far as we know, this is a retrospective study with the largest cohort of ENB with orbital invasion thus far within a single institution. Several interesting and new findings were discovered from this report. First, the prognostic value of distinction between invasion of the bony orbit and deeper soft tissue was found to exist in ENB. Second, the survival rate after radical radiation was comparable to the combination of surgery and radiotherapy among patients with aggressive tumors and stages (Foote stage of C/D). Third, no patient was managed with orbital exenteration, even in cases that extended to extraocular muscles or the eye globe.

There is no standardized staging system for ENB, and many staging systems have been proposed for ENB. The Kadish staging system [2] is still the most popular system because of its simplicity and ease of application. According to the Kadish system, tumors that extend beyond the nose and paranasal sinuses are designated as stage C [2]. Therefore, the system recognizes orbital invasion as a relatively negative prognostic factor, given that tumors with orbital invasion are classified as stage C. However, there are many criticisms of the Kadish system, and the prognosis for each stage is inconsistent among studies [13] because the details of local extension of the tumor were not assessed. Joshi et al. [14] used the National Cancer Database to evaluate the prognostic ability of the Kadish staging system. This multivariate analysis controlling for stage failed to demonstrate clear survival differences between stages in the Kadish system, which poorly predicted prognosis over a 10-year horizon. The prognostic value of the distinction between invasion of the bony orbit and deeper soft tissue had been reflected in the AJCC staging categories, which divide bone orbital wall invasion (T3) from invasion of the anterior two-thirds (T4a) and orbit apex (T4b) for sinonasal tumors of epithelial origin [3, 4]. In our study, we followed the classification method proposed by Iannetti [12] (stated in Material and Methods). Using this classification method, a univariate analysis showed that grade I orbital invasion was associated with better OS and PFS than was grade II/III. The outcome means that deeper orbital invasion is a negative prognostic factor in ENB. To our surprise, there was no significant difference between grade II and grade III in survival rate, and the 5-year OS of patients with involvement of the orbital apex was comparable to that of patients without involvement (62.3% vs. 70.2%, *p* = 0.173). Based on this observation, we believe that the Iannetti classification method could be a good reference for clinical treatment, but a long-term additional study with a larger sample size should be conducted to help determine whether orbital invasion is an independent prognosis factor in ENB.

The treatment strategy of ENB remains controversial. Due to the rarity of these tumors and the lack of prospective randomized controlled clinical trials, there is no generally accepted therapeutic regimen for ENB [15–17]. Comprehensive treatment is usually recommended, as the majority of cases present with advanced ENB, and surgical resection followed by postoperative radiotherapy demonstrated better treatment outcomes with 5-year OS rates of 60–88% in previous retrospective reports [18–20], compared to radiotherapy alone (29–54%) [15, 19, 21, 22]. Although previous reports indicated that comprehensive treatment was superior to radical radiotherapy, the results should be interpreted with caution, because patients with wider infringement of lesions may be given radical radiotherapy rather than surgery. Therefore, there was a significant case selection bias between various treatment modalities [15, 19]. The clinical data of 21 patients with T4 ENB treated with C-ion radiotherapy were retrospectively reviewed by Suefji [23]. The 3-year overall survival and local control rate were 88.4 and 83%, respectively. Nakamura et al. [24] described 42 consecutive patients who received proton beam therapy with curative intent for ENB. Twenty-eight (67%) patients had Kadish stage C and achieved a 5-year OS of 76%. In our study, 7 (50%) patients had a complete response among the definitive radiotherapy group. More interestingly, the abnormal signals of residual tumors which disappeared spontaneously during follow-up were found in 3 patients with PR. Radical radiotherapy still achieved a 5-year OS of 63.5%, which was higher than that of surgery plus postoperative radiation (58.2%). These results suggest that radical radiotherapy is not merely palliative but has therapeutic efficacy as a primary treatment for a notable percentage of locally advanced or unresectable ENBs.

For Kadish/Foote stage C tumors, there is little consensus regarding the timing of radiotherapy for a combination of surgery and radiation. The complex anatomical location, as well as visual and facial structures, is “challenging” in terms of performing radical surgery while preserving functional organs and improving local control rates. Only a few studies have been reported in terms of preoperative radiation in the treatment of sinonasal cancers. Erik et al. [25] reported 79 patients with sinonasal cancer who were treated with preoperative radiation, and orbital exenteration was performed in 7% of these patients. A total of 11,160 sinonasal malignancy patients were identified from the National Cancer Data Base for analysis by Robin [26]. The findings were validated in propensity score matching, and it is important to note that neoadjuvant chemoradiotherapy was associated with achieving a negative surgical margin and improved OS. For ENB, Polin et al. [27] showed significant tumor reduction in two-thirds of patients treated with preoperative radiation with or without chemotherapy and increased recurrence-free survival in those responders. Sohrabi et al. [28] reported complete pathologic responses in two stage C ENB patients treated with neoadjuvant concurrent radiochemotherapy. In our series, preoperative radiotherapy yielded the most promising results, including a higher long-term survival, than did postoperative radiotherapy (5-year OS 79% for preoperative radiation vs. 58.2% for postoperative radiation, *p* = 0.215). Considering the high residual rate of primary surgery (76%), we believe that preoperative radiation not only improves the tumor resection rate but also increases the potential for orbital preservation surgery by reducing tumor mass and eliminating microscopic lesions at the periphery of the tumor. The treatment strategy of preoperative radiotherapy and/or chemotherapy followed by surgery for Kadish stage C/D disease was also advocated for by a European position paper on endoscopic management of tumors of the nose, paranasal sinuses and skull [29].

The indications for orbital exenteration are continuously changing, from invasion of orbital periosteum [30] to beyond the periosteum [6], to extraconal fat [10], and extraconal muscles [9]. Imola and Schramm [10] reported 66 patients in which the orbit was preserved, even in cases of limited involvement of extraconal fat. Orbital exenteration was performed in 12 cases, and similar survival rates were achieved in cases of orbital preservation and exenteration. In a cohort reported on by Lisan et al. [9], orbital preservation was undertaken in 66% of cases, whereas orbital exenteration was performed in cases of invasion of the extraocular muscles, ocular globe, or orbital apex. Local control rates and 5-year survival were similar in both groups. In previous studies, which include several pathological types [9–11, 25] with varying prognoses, the evaluation of orbital invasion was ill defined [11, 25]. In our study, the histology was limited to ENB. Due to the good response to radiotherapy, no patient underwent orbital exenteration. The 5-year OS was 69% among all cases, which is consistent with the rates of 65–75% reported in previous studies [18–20]. These results suggested that orbital preservation did not lead to poor survival rate, and invasion of extraocular muscles or the eye globe was not a contraindication for eye-sparing surgery in ENB.

In our study, lymph node status is the important prognostic factor affecting OS, DMFS, and PFS. Regarding 8 patients with N+, 7 patients underwent elective neck irradiation with no regional recurrence. According to this observation, we consider that patients in stage D disease with LN metastasis require only irradiation to the neck instead of neck dissection. Regarding patients with N0, ENB has a high rate of late regional recurrence (20–44%) [31, 32]. However, the value of prophylactic elective neck irradiation (PENI) for patients with N0 neck status is still unclear. In a large cohort (116 patients) reported by Yin et al. [33], PENI was an independent favorable predictor for regional controlling, and the author recommended PENI as a part of initial treatment strategy for patients with Foote stage B/C tumors. Two other studies hold a differing opinion that PENI plays a limited role in improving regional control [34, 35]. Our study showed that PENI improved the LRFS significantly from 58.1 to 100% (*p* = 0.004). Although the *p*- value was not statistically significant, the 5-year OS was still higher in patients treated with PENI than patients not treated with PENI.

Although patients who received chemotherapy had better LRFS compared to those who received treatment without chemotherapy in our study, the therapeutic effects of chemotherapy on ENB cannot be determined for the reason that the cohort lacked a standard chemotherapy protocol that was uniformly applied. Further clinical studies are needed to evaluate the value of chemotherapy for ENB. Our study had several other limitations. It was a retrospective analysis based on data from a single center. The Hyams grading system [36] that can be used as a prognostic indicator for ENB was not assessed in our study. A multicenter clinical trial is indispensable for further investigation.

## Conclusion

Our data suggest that orbital invasion in grade II/III was significantly associated with adverse survival outcomes. Invasion of either the extraocular muscles or eye globe is not a contraindication for eye-sparing surgery. Primary radiotherapy achieved comparable survival to surgery plus radiotherapy in advanced and unresectable ENB. Prophylactic radiotherapy to the neck in patients with N0 significantly reduces the risk of regional recurrence.

## Data Availability

The datasets used and/or analysed during the current study are available from the corresponding author on reasonable request.

## Abbreviations

3D-CRT: Three-dimensional conformal radiotherapy
AJCC: American Joint Committee on Cancer
CR: Complete response
CTV: Clinical target volume
DMFS: Distant metastasis–free survival
ENB: Esthesioneuroblastoma
GTV: Gross tumor volume
HR: Hazard ratio
IMRT: Intensity-modulated radiotherapy
LRFS: Locoregional relapse-free survival
OS: Overall survival
PD: Progression disease
PENI: Prophylactic elective neck irradiation
PFS: Progression-free survival
PR: Partial response
PTV: Planning target volume
SD: Stable disease
TENI: Therapeutic elective neck irradiation
